# Understanding primary care providers’ attitudes towards preventive screenings to patients with inflammatory bowel disease

**DOI:** 10.1371/journal.pone.0299890

**Published:** 2024-04-25

**Authors:** Fang Xu, Susan A. Carlson, Kurt J. Greenlund

**Affiliations:** Division of Population Health, National Center for Chronic Disease Prevention and Health Promotion, Centers for Disease Control and Prevention, Atlanta, Georgia, United States of America; Center for Primary Care and Public Health, SWITZERLAND

## Abstract

**Background:**

Preventive care is important for managing inflammatory bowel disease (IBD), yet primary care providers (PCPs) often face challenges in delivering such care due to discomfort and unfamiliarity with IBD-specific guidelines. This study aims to assess PCPs’ attitudes towards, and practices in, providing preventive screenings for IBD patients, highlighting areas for improvement in guideline dissemination and education.

**Methods:**

Using a web-based opt-in panel of PCPs (DocStyles survey, spring 2022), we assessed PCPs’ comfort level with providing/recommending screenings and the reasons PCPs felt uncomfortable (*n* = 1,503). Being likely to provide/recommend screenings for depression/anxiety, skin cancer, osteoporosis, and cervical cancer were compared by PCPs’ comfort level and frequency of seeing patients with IBD. We estimated adjusted odd ratios (AORs) of being likely to recommend screenings and selecting responses aligned with IBD-specific guidelines by use of clinical practice methods.

**Results:**

About 72% of PCPs reported being comfortable recommending screenings to patients with IBD. The top reason identified for not feeling comfortable was unfamiliarity with IBD-specific screening guidelines (55%). Being comfortable was significantly associated with being likely to provide/recommend depression/anxiety (AOR = 3.99) and skin cancer screenings (AOR = 3.19) compared to being uncomfortable or unsure. Percentages of responses aligned with IBD-specific guidelines were lower than those aligned with general population guidelines for osteoporosis (21.7% vs. 27.8%) and cervical cancer screenings (34.9% vs. 43.9%), and responses aligned with IBD-specific guidelines did not differ by comfort level for both screenings. Timely review of guidelines specific to immunosuppressed patients was associated with being likely to provide/recommend screenings and selecting responses aligned with IBD-specific guidelines.

**Conclusions:**

Despite a general comfort among PCPs in recommending preventive screenings for IBD patients, gaps in knowledge regarding IBD-specific screening guidelines persist. Enhancing awareness and understanding of these guidelines through targeted education and resource provision may bridge this gap.

## Introduction

Inflammatory bowel disease (IBD), including Crohn’s disease and ulcerative colitis, characterizes chronic inflammation of the gastrointestinal tract. In 2015 and 2016, 3.1 million US adults reported having either Crohn’s disease or ulcerative colitis [1]. As IBD is usually diagnosed in one’s 20s and 30s [2], patients with IBD need to manage their condition for decades, which requires substantial health care costs in the US health care system [3]. Patients with IBD, vs. those without, are also more likely to have comorbidities and certain cancers [4]. Therefore, timely and appropriate preventive screening among patients with IBD is an essential component to overall disease management, which is recommended by the American College of Gastroenterology (ACG) [5].

Screening practices for adults with IBD may differ when compared to those for the general population. IBD-specific screening guidelines for depression, anxiety, skin cancer, osteoporosis, and cervical cancer from the ACG [5] are compared to those from the United States Preventive Services Task Force (USPSTF) recommendations for the general population in Table 1. The ACG guideline and the USPSTF recommendation for depression screening are similar [6]. The USPSTF does not currently have a recommendation about screening for anxiety in adults; however, the draft USPSTF recommendation for screening for anxiety in adults is similar to that in the ACG guideline [7]. The ACG guideline recommends melanoma skin cancer screening for patients with IBD as well as nonmelanoma skin cancer screening for those on immunomodulators, while the USPSTF states that evidence is insufficient to recommend skin cancer screening for the general adult population [8]. For cervical cancer screening, the ACG guideline—compared with USPSTF recommendation—recommends more frequent screening for women with IBD on immunosuppressive therapy [9]. Finally, the ACG guideline recommends patients with conventional risk factors be screened for osteoporosis at the time of IBD diagnosis and periodically thereafter, which differs from the USPSTF recommendation that only women aged ≥ 65 years, or age < 65 years who are postmenopausal and at increased risk for osteoporosis, should undergo the screening [10].

**Table 1:** Screening guidelines for depression and anxiety, skin cancer, osteoporosis, and cervical cancer for the general population and patients with IBD.

Topic	General population^a^	Patients with IBD^b^
Depression and anxiety	Screening for depression in the adult population, including pregnant and postpartum women, as well as older adults (Final, 2023, *Grade B*).^c^ Screening anxiety disorders in adults 64 years or younger, including pregnant and postpartum persons (*Grade B*); the current evidence is insufficient to assess the balance of benefits and harms of screening for anxiety disorders in adults aged 65 years or older (Final, 2023, *Grade I*).^d^	Screening for depression and anxiety is recommended in patients with IBD (*Conditional recommendation*,^e^ *low level evidence*).
Skin cancer	Current evidence is insufficient to assess the balance of benefits and harms of visual skin examination by a clinician to screen for skin cancer in adults (2016, *Grade I*).^f^	Patients with IBD should undergo screening for melanoma independent of the use of biologic therapy (*Strong recommendation*, *low level of evidence*). IBD patients on immunomodulators should undergo screening for nonmelanoma squamous cell cancer while using these agents, particularly over the age of 50 (*Strong recommendation*, *low level of evidence*).
Cervical cancer	Screening for cervical cancer every 3 years with cervical cytology alone in women aged 21 to 29 years (*Grade A*).^g^ For women aged 30 to 65 years, screening every 3 years with cervical cytology alone, every 5 years with high-risk human papillomavirus (hrHPV) testing alone, or every 5 years with hrHPV testing in combination with cytology (cotesting) (2018, *Grade A*).^g^	Women with IBD on immunosuppressive therapy should undergo annual cervical cancer screening (*Conditional recommendation*, *very low level evidence*).
Osteoporosis	Screening for osteoporosis with bone measurement testing to prevent osteoporotic fractures in women 65 years and older (*Grade B*).^h^ Screening for osteoporosis with bone measurement testing to prevent osteoporotic fractures in postmenopausal women younger than 65 years who are at increased risk of osteoporosis as determined by a formal clinical risk assessment tool (2018, *Grade B*).^h^	Patients with conventional risk factors for abnormal bone mineral density with IBD should undergo screening for osteoporosis with bone mineral density testing at the time of [IBD] diagnosis and periodically after diagnosis (*Conditional recommendation*, *very low level of evidence*).

Abbreviation: IBD, inflammatory bowel disease.

^a^United States Preventive Services Task Force.

^b^Farraye FA, Melmed GY, Lichtenstein GR, Kane SV. ACG clinical guideline: Preventive care in inflammatory bowel disease. *Am J Gastroenterol*. 2017 Feb;112(2):241–58. doi: 10.1038/ajg.2016.537

^c^United States Preventive Services Task Force. Depression and suicide risk in adults: Screening (June 20, 2023). Available at: https://www.uspreventiveservicestaskforce.org/uspstf/recommendation/screening-depression-suicide-risk-adults.

^d^United States Preventive Services Task Force. Anxiety disorders in adults: Screening (June 20, 2023). Available at: https://www.uspreventiveservicestaskforce.org/uspstf/recommendation/anxiety-adults-screening.

^e^The American College of Gastroenterology graded recommendations as “conditional” when there was uncertainty about the tradeoffs between the desirable and undesirable effects of an intervention.

^f^United States Preventive Services Task Force. Skin cancer: Screening (April 18, 2023). Available at: https://www.uspreventiveservicestaskforce.org/uspstf/recommendation/skin-cancer-screening.

^g^United States Preventive Services Task Force. Cervical cancer: Screening (August 21, 2018). Available at: https://www.uspreventiveservicestaskforce.org/uspstf/recommendation/cervical-cancer-screening.

^h^United States Preventive Services Task Force. Osteoporosis to prevent fractures: Screening (June 26, 2018). Available at: https://www.uspreventiveservicestaskforce.org/uspstf/recommendation/osteoporosis-screening.

Previous studies have found mixed results when examining the rate of receiving general preventive care among adults with IBD vs. those without [11,12]. One study used nationally representative data and found the prevalence of general preventive care services (e.g., blood pressure screening, cholesterol check, colorectal cancer screening) was more common among adults with IBD than those without [12]. Another study used a cross-sectional analysis of IBD patients and controls and concluded that IBD patients did not receive preventive services (measure using an index of 10 common preventative services) at the same rate as patients in a general medical practice [11]. These different findings may be due to differing study periods, settings, sample sizes, and statistical approaches.

Primary care providers (PCPs) play an important role providing or recommending preventive screenings [13]; however, primary care physicians may not feel comfortable providing preventive care to patients with IBD [14]. In addition, it is not clear who should recommend these screenings to patients with IBD—PCPs or gastroenterologists [14]. Vaccination is an important aspect of preventive screening for patients with IBD. We previously assessed PCPs’ attitudes towards vaccinations recommendations for patients with IBD and identified practices that may help support PCP immunization practices for these patients [15]. For the purpose of this study, we evaluated PCP’s attitudes and practices related to preventive screening and how these align with IBD-specific guidelines assessing whether PCP practices align with IBD-specific guidelines. Such information can help identify opportunities to improve preventive screening practices for patients with IBD.

There are four study objectives. We examined information from a national opt-in panel of PCPs to assess 1) PCPs’ overall level of comfort recommending or providing preventive screenings by PCP characteristics, 2) reasons PCPs report for not feeling comfortable recommending or providing preventive screenings, 3) PCPs’ likelihood of offering or recommending screenings for depression, anxiety and skin cancer, as well as PCPs’ practices regarding screenings for osteoporosis and cervical cancer, and 4) clinical practice methods used for screening immunosuppressed patients (e.g., IBD). These data were used to examine differences in PCPs’ overall comfort with recommending or providing preventive screenings by their characteristics, associations between PCPs’ comfort level or clinical practice methods and likelihood of recommending or providing depression or anxiety and skin cancer screening, and whether PCPs’ responses aligned with osteoporosis and cervical cancer screening guidelines for patients with IBD.

## Materials and methods

### Study sample

We designed a cross-sectional study by including questions about preventive screenings for patients with IBD in the DocStyles survey conducted in spring 2022. DocStyles is a web-based survey conducted by Porter Novelli Public Services to assess healthcare providers’ attitudes and knowledge regarding clinical practice. The sample frame was active US members of Sermo’s Global Medical Panel [16], in which 350,000 US health care providers enrolled. Centers for Disease Control and Prevention (CDC) has a license agreement with Porter Novelli on DocStyles 2022 which included questions regarding IBD.

A total of 1,503 respondents who practiced in the United States for at least 3 years and actively saw patients in an individual or group outpatient practice, or inpatient practice completed the survey. Respondents were paid an honorarium based on the number of questions asked of their specialty. The study sample included 1,000 primary care physicians (family practitioners and internists), 253 obstetricians or gynecologists (OB-GYNs), and 250 nurse practitioners or physician assistants. Although pediatricians participated in the DocStyles survey, they were not asked the questions about preventive screenings for patients with IBD since the focus was preventive care for adults.

### Survey questions

There were 8 questions assessing PCPs’ attitudes and practices towards preventive screenings for patients with IBD. The first question asked about PCPs’ frequency (within the past 6 months, more than 6 months ago, never, or unsure) of seeing patients with IBD (Table 2). The second question assessed PCPs’ comfort recommending or providing preventive screenings to patients with IBD, whether or not they currently saw such patients. If PCPs answered “uncomfortable” or “unsure,” they were asked a follow-up question about the reason and could select all that apply from the following list: “not familiar with IBD medications or patients’ immunosuppressive status,” “not familiar with screening guidelines for patients with IBD,” “prefer multidisciplinary teamwork on preventive screenings for patients with IBD,” and “other reasons.” The next 2 questions asked about PCPs’ likelihood of providing or recommending screenings for depression or anxiety and skin cancer to patients with IBD, using a Likert scale response (very likely, likely, neither likely nor unlikely, unlikely, and very unlikely). The sixth question asked when PCPs would recommend patients with IBD—who have conventional risk factors for abnormal bone mineral density—receive an osteoporosis screening with bone mineral density testing. The responses included “female patients at age of 65 years or older,” “female patients at time of IBD diagnosis,” “female patients at time of IBD diagnosis and periodically after diagnosis,” “all patients at time of IBD diagnosis,” “all patients at time of IBD diagnosis and periodically after diagnosis,” and “not sure.” The seventh question assessed cervical cancer screening by assessing how frequently PCPs would recommend Pap tests be initially done (i.e., prior to consecutive normal test results) for women with IBD on immunosuppressive therapy. The responses included “once every 5 years,” “once every 3 years,” “once every 1 year,” “none of these,” and “not sure.” The last question assessed methods PCPs’ practices use for screening immunosuppressed patients (e.g., IBD). The responses included “a timely review of preventive care guidelines specific to immunosuppressed patients,” “decision support using patients’ electronic health records alerts,” “clinical risk assessment tools,” and “other methods”; respondents could select all that apply.

**Table 2:** Survey questions related to primary care providers’ attitudes towards preventive screenings among patients with IBD, 2022 Spring DocStyles.

Question #	Questions regarding preventive screenings to patients with IBD
Q1	In your practice, have you seen patients with inflammatory bowel disease (IBD), a disease which mainly includes Crohn’s disease and ulcerative colitis? Yes, within the past 6 months Yes, more than 6 months ago Never Unsure
Q2	The following questions are related to your attitudes towards recommending or providing preventive screenings for patients with IBD. Please answer these questions whether or not you currently see patients with IBD. Are you comfortable or uncomfortable recommending or providing preventive screenings to patients with IBD? Comfortable Uncomfortable Unsure
Q3 (select all that apply, skipped by PCPs who answered 1 to Q2)	What are the reasons that you are unsure or uncomfortable? Not familiar with IBD medications or patients’ immunosuppressive status Not familiar with screening guidelines for patients with IBD Prefer multidisciplinary teamwork on preventive screenings for patients with IBD Other reasons
Q4	How likely would you be to provide or recommend a screening for depression and anxiety to patients with IBD? Very likely Likely Neither likely nor unlikely Unlikely Very unlikely
Q5	How likely would you be to provide or recommend a screening for skin cancer to patients with IBD? Very likely Likely Neither likely nor unlikely Unlikely Very unlikely
Q6	When would you recommend that patients with IBD who have conventional risk factors for abnormal bone mineral density receive an osteoporosis screening with bone mineral density testing? Female patients at age 65 years or older Female patients at time of IBD diagnosis Female patients at time of IBD diagnosis and periodically after diagnosis All patients at time of IBD diagnosis All patients at time of IBD diagnosis and periodically after diagnosis Not sure
Q7	How frequently would you recommend Pap tests be initially done (i.e., prior to consecutive normal test results) for women with IBD on immunosuppressive therapy? Once every 5 years Once every 3 years Once every 1 year None of these Not sure
Q8 (select all that apply)	Which method(s) does your practice use for screening immunosuppressed patients (e.g., IBD)? A timely review of preventive care guidelines specific to immunosuppressed patients Decision support using patients’ electronic health records alerts Clinical risk assessment tools Other methods

Abbreviation: IBD, inflammatory bowel disease.

The survey also collected characteristics about the PCPs, including age (25‒39, 40‒49, and ≥ 50 years), sex, race/ethnicity (non-Hispanic White, non-Hispanic Asian, and other), medical specialty (family practitioner, internist, OB-GYN, and nurse practitioner or physician assistant), years of practice (< 15 and ≥ 15 years), average number of patients seen per week (1–50, 51–100, and ≥ 101), main work setting (individual outpatient practice, group outpatient clinic or practice, and inpatient practice or hospital), hospital teaching status (teaching and non-teaching), worksite location (urban, suburban, and rural), region of practice (Northeast, Midwest, South, and West), and majority of patients’ household income (< $50,000, $50,000 to < $100,000, and ≥ $100,000).

### Statistical analyses

We calculated percentages of PCPs’ level of comfort recommending or providing preventive screenings to patients with IBD by PCP characteristics. We used the Cochran-Mantel-Haenszel test to compare differences by levels of comfort. For PCPs who reported being uncomfortable or unsure recommending or providing preventive screenings to patients with IBD, we calculated percentages for the reasons they identified. Next, we calculated percentages and estimated odds of PCPs being likely or very likely to provide or recommend screenings for depression or anxiety and skin cancer by level of comfort and by frequency of seeing patients with IBD in practice. We then calculated percentages of PCPs’ responses to the questions about screenings for osteoporosis (who should be screened) and cervical cancer (frequency of Pap tests)—by level of comfort and frequency of seeing patients with IBD in practice. Lastly, we estimated adjusted odds of 1) PCPs being likely or very likely to provide or recommend screenings for depression, anxiety and skin cancer, and 2) PCPs’ responses aligning with the ACG guideline for osteoporosis and cervical cancer specific to patients with IBD by approaches used by PCPs’ practice for screening immunosuppressed (e.g., IBD) patients. χ^2^ statistics were used for group comparison, and models were adjusted for feeling comfortable with recommending or providing preventive screenings to patients IBD, frequency of seeing such patients, medical specialty, years of practice, and average number of patients seen per week. We conducted the analyses using the entire study sample and a supplementary analysis limited to family practitioners and internists. The significance level was set at 0.05. The analyses were performed using SAS 9.4 (SAS Institute, Research Triangle, Cary, North Carolina). The study was deemed to be exempt from the Centers for Disease Control and Prevention (CDC) Institutional Review Board because the database shared with CDC did not include personal identifiers.

## Results

Of the 1,503 PCPs surveyed, two-thirds were family practitioners or internists, 17% were OB-GYNs, and 17% were nurse practitioners or physician assistants. About 60% were younger than 50 years, 59% were men, 63% were non-Hispanic White persons, two-thirds were in a group outpatient clinic or practice, 9% practiced in rural areas, and one-third had a majority of patients with household income < $50,000 (Table 3).

**Table 3:** Primary care providers reported level of comfort recommending or providing preventive screenings to patients with IBD by primary care providers’ demographic and clinical characteristics (*N* = 1,503).

Primary care provider characteristics^a^	Overall %	Primary care providers’ level of comfort recommending or providing preventive screenings to patients with IBD			*P* value^b^
		Comfortable % (95% CI)	Unsure % (95% CI)	Uncomfortable % (95% CI)	
**All**	100	71.9 (70.7–74.2)	10.7 (9.1–12.2)	17.4 (15.5–19.4)	
**Medical specialty**					<0.001
Family practitioner	32.4	81.1 (77.6–84.6)	9.0 (6.5–11.6)	9.9 (7.2–12.5)	
Internist	34.2	77.6 (74.0–81.2)	8.8 (6.3–11.2)	13.6 (10.7–16.6)	
Obstetrician or gynecologist	16.8	53.4 (47.2–59.5)	13.4 (9.2–17.6)	33.2 (27.4–39.0)	
Nurse practitioner or physician assistant	16.6	61.2 (55.2–67.2)	14.8 (10.4–19.2)	24.0 (18.7–29.3)	
**Age** (yrs)					0.74
25–39	33.5	72.8 (68.9–76.7)	9.3 (6.8–11.9)	17.9 (14.5–21.2)	
40–49	27.3	70.1 (65.8–74.7)	11.0 (8.0–14.0)	18.8 (15.0–22.6)	
≥ 50	39.2	72.4 (68.8–76.0)	11.5 (9.0–14.1)	16.1 (13.1–19.1)	
**Sex**					0.01
Male	59.3	74.5 (71.6–77.4)	9.8 (7.8–11.7)	15.7 (13.3–18.1)	
Female	40.7	68.7 (64.4–71.9)	11.9 (9.3–14.5)	20.0 (16.8–23.2)	
**Race**					0.08
Non-Hispanic White	63.2	69.8 (66.9–72.7)	11.5 (9.5–13.5)	18.7 (16.3–21.2)	
Non-Hispanic Asian	20.8	76.0 (71.3–80.8)	8.0 (5.0–11.0)	16.0 (11.9–20.0)	
Other^c^	16.0	75.0 (69.5–80.5)	10.8 (6.9–14.8)	14.2 (9.8–18.6)	
**Years of practice**					0.69
< 15	48.7	71.6 (68.3–74.9)	10.5 (8.3–12.7)	17.9 (15.1–20.7)	
≥ 15	51.3	72.2 (69.1–75.4)	10.8 (8.6–13.0)	17.0 (14.3–19.6)	
**Average number of all patients seen per week**					<0.001
1–50	16.8	59.7 (53.6–65.7)	12.6 (8.6–16.7)	27.7 (22.2–33.2)	
51–100	52.6	72.5 (69.4–75.6)	10.6 (8.5–12.8)	16.8 (14.2–19.4)	
≥ 101	30.6	77.6 (73.8–81.4)	9.6 (6.9–12.3)	12.8 (9.8–15.9)	
**Main work setting**					0.49
Individual outpatient practice	17.0	71.8 (66.2–77.3)	9.0 (5.5–12.5)	19.2 (14.4–24.1)	
Group outpatient clinic or practice	64.5	72.7 (69.9–75.5)	10.8 (8.9–12.8)	16.5 (14.2–18.8)	
Inpatient practice or hospital	18.5	69.4 (64.0–74.8)	11.5 (7.8–15.3)	19.1 (14.5–23.7)	
**Hospital teaching status**					0.05
Teaching	48.8	74.8 (71.7–77.9)	8.9 (6.8–10.9)	16.3 (13.7–19.0)	
Non-teaching	51.2	69.2 (65.9–72.4)	12.3 (10.0–14.7)	18.5 (15.7–21.2)	
**Worksite**					0.24
Urban	36.9	71.2 (67.4–74.9)	8.8 (6.5–11.2)	20.0 (16.7–23.3)	
Suburban	53.7	71.5 (68.4–74.6)	12.3 (10.0–14.5)	16.2 (13.7–18.8)	
Rural	9.4	77.3 (70.4–84.2)	8.5 (3.9–13.1)	14.2 (8.4–19.9)	
**US region**					0.55
Northeast	24.1	70.7 (66.0–75.4)	12.9 (8.8–15.5)	17.1 (13.3–21.0)	
Midwest	22.9	72.7 (68.0–77.4)	12.2 (8.8–15.7)	15.1 (11.3–18.9)	
South	32.5	74.0 (70.1–77.9)	8.2 (5.8–10.6)	17.8 (14.4–21.2)	
West	20.5	69.3 (64.1–74.4)	11.0 (7.5–14.5)	19.7 (15.3–24.2)	
**Patients’ household income**					0.86
Poor (<$25,000) or lower middle ($25,000 to <$50,000)	32.3	72.6 (68.7–76.6)	9.3 (6.7–11.8)	18.1 (14.7–21.5)	
Middle ($50,000 to <$100,000)	41.9	71.4 (67.9–75.0)	11.0 (8.5–13.4)	17.6 (14.6–20.6)	
Upper middle ($100,000 to <$250,000) or affluent (≥$250,000)	25.8	71.8 (67.4–76.3)	11.9 (8.7–15.1)	16.3 (12.6–20.0)	
**Frequency of seeing patients with IBD in practice**					<0.001
Within the past 6 months	79.6	78.7 (76.4–81.0)	8.5 (7.8–10.1)	12.8 (10.9–14.7)	
More than 6 months ago	15.4	53.9 (47.5–60.3)	14.2 (9.7–18.7)	31.9 (25.9–37.9)	
Never or not sure	5.0	20.0 (11.0–29.1)	33.3 (22.7–44.0)	46.7 (35.4–58.0)	

Abbreviations: IBD, inflammatory bowel disease; CI, confidence interval.

^a^The survey includes primary care providers who live and work in the United States and actively see patients.

^b^Based on Cochran-Mantel-Haenszel test.

^c^The “other race” category includes Hispanic, non-Hispanic Black, Native Hawaiian or Pacific Islander, American Indian or Alaska Native, or other races or ethnicities.

Overall, 72% PCPs reported they were comfortable recommending or providing preventive screenings to patients with IBD (Table 3). Male PCPs and family practitioners or internists were more likely to report being comfortable recommending or providing preventive screenings to patients with IBD (thereafter mentioned as “report being comfortable”) compared with women, OB-GYNs, and nurse practitioners or physician assistants. PCPs who saw the most patients (average ≥ 101 per week) were most likely to report being comfortable, while PCPs who saw the fewest patients (1‒50 per week averagely) were least likely to report being comfortable. PCPs who saw patients with IBD in their practice frequently (within in the past 6 months) were most likely to report being comfortable, while PCPs who never saw patients with IBD or were unsure were least likely to report being comfortable.

**Fig 1:**
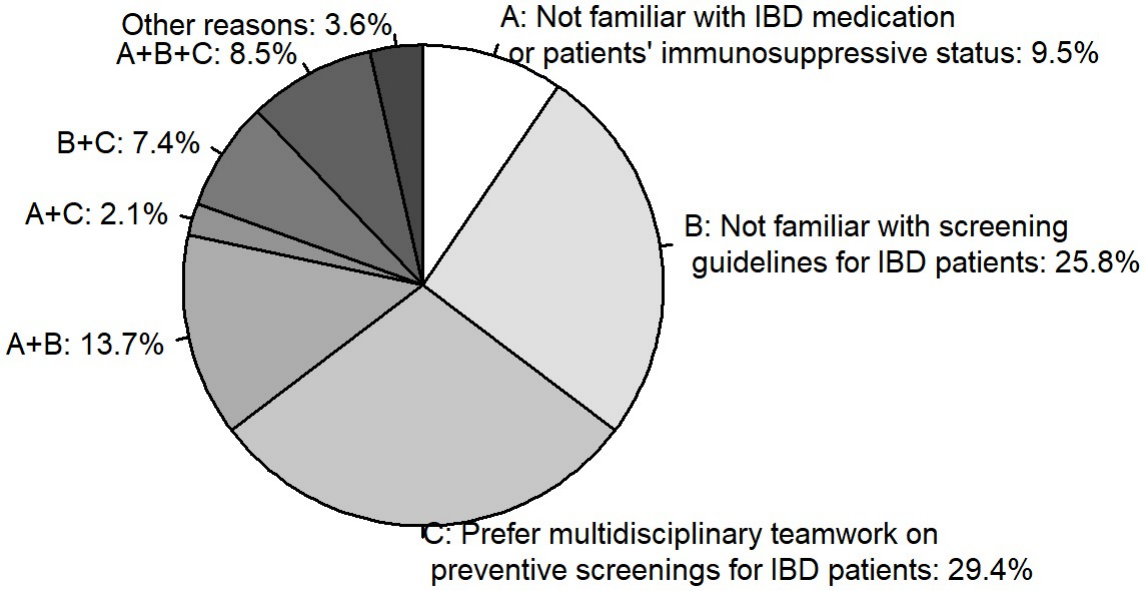
Reasons identified by PCPs who are uncomfortable or unsure of recommending or providing preventive screenings to patients with IBD, DocStyles spring 2022 (*n* = 422)^a^. Abbreviations: IBD, inflammatory bowel disease; PCP, primary care provider. ^a^The multiple-choice question, “What are the reasons that you are unsure or uncomfortable?” applied only to respondents who answered “uncomfortable” or “unsure” to the question, “Are you comfortable or uncomfortable recommending or providing preventive screenings to patients with IBD?”.

Of the 422 PCPs who reported being uncomfortable or unsure, 55% reported that they were not familiar with screening guidelines for patients with IBD, 47% reported preferring multidisciplinary teamwork for preventive screenings for patients with IBD, and 34% reported they were not familiar with IBD medications or patients’ immunosuppressive status (Fig 1). Of these PCPs, 65% identified 1 of these 3 reasons, 23% identified 2 reasons, 8% identified all 3 reasons, and 4% identified other reasons.

About 79% of PCPs were likely (39% very likely, 40% likely) to provide or recommend screenings for depression and anxiety to patients with IBD, while 56% were likely (23% very likely, 33% likely) to provide or recommend screenings for skin cancer (Table 4). Factors associated with providing or recommending screening for depression, anxiety and skin cancer included PCPs reporting being comfortable vs. being uncomfortable or unsure. Frequency of seeing patients with IBD was significantly associated with providing or recommending depression, anxiety screening, but not skin cancer screening.

**Table 4:** Percentages and adjusted odds ratios of PCPs’ likelihood^a^ to provide or recommend screenings for depression, anxiety and skin cancer by primary care providers’ comfort level, and frequency of seeing patients with IBD in practice/seeing patients with IBD in practice.

Select characteristics	All (*N* = 1,503) %	Likely to provide or recommend screenings for			
		Depression and anxiety		Skin cancer	
		%^b^ (95% CI)	AOR (95% CI)	%^b^ (95% CI)	AOR (95% CI)
**All**	100	78.9 (76.9−81.0)	—	55.7 (53.2−58.2)	—
**Reported level of comfort recommending or providing preventive screenings** ^c^					
Comfortable	71.9	86.5*** (84.5−88.5)	3.99*** (2.87−5.54)	63.7*** (60.9−66.6)	3.19*** (2.34−4.34)
Unsure	10.7	65.0 (57.6−72.4)	1.39 (0.91−2.13)	42.5* (34.8−50.2)	1.58* (1.04−2.39)
Uncomfortable (ref)	17.4	56.1 (50.1−62.1)	1	30.5 (25.0−36.1)	1
**Reported frequency of seeing IBD patients in practice** ^d^					
Within the past 6 months	79.6	82.9*** (80.8−85.1)	2.91*** (1.73−4.90)	58.7*** (55.9−61.5)	1.59 (0.92−2.74)
More than 6 months ago	15.4	69.0*** (63.0−74.9)	1.86* (1.05−3.30)	47.8* (41.4−54.3)	1.42 (0.79−2.53)
Never or not sure (ref)	5.0	45.3 (34.1−56.7)	1	32.0 (21.4− 42.6)	1

* 0.01 < *P* ≤ 0.05

** 0.001 < *P* ≤ 0.01

*** *P* < 0.001.

Abbreviations: PCPs, primary care providers; AOR, adjusted odds ratio; CI, confidence interval; IBD, inflammatory bowel disease; ref, referent group.

^a^ PCPs were asked how likely they were to provide or recommend a screening for “depression and anxiety” or “skin cancer” to patients with IBD. Responses were “very likely” or “likely” vs. “neither likely nor unlikely,” “unlikely,” or “very unlikely.”

^b^ Percentage comparisons are based on χ^2^ test.

^c^The multivariable logistic regressions adjusted for frequency of seeing IBD patients, medical specialty, years of practice, and average number of patients seen per week.

^d^The multivariable logistic regressions adjusted for being comfortable recommending or providing preventive screenings to IBD patients, medical specialty, years of practice, and average number of patients seen per week.

**Fig 2:**
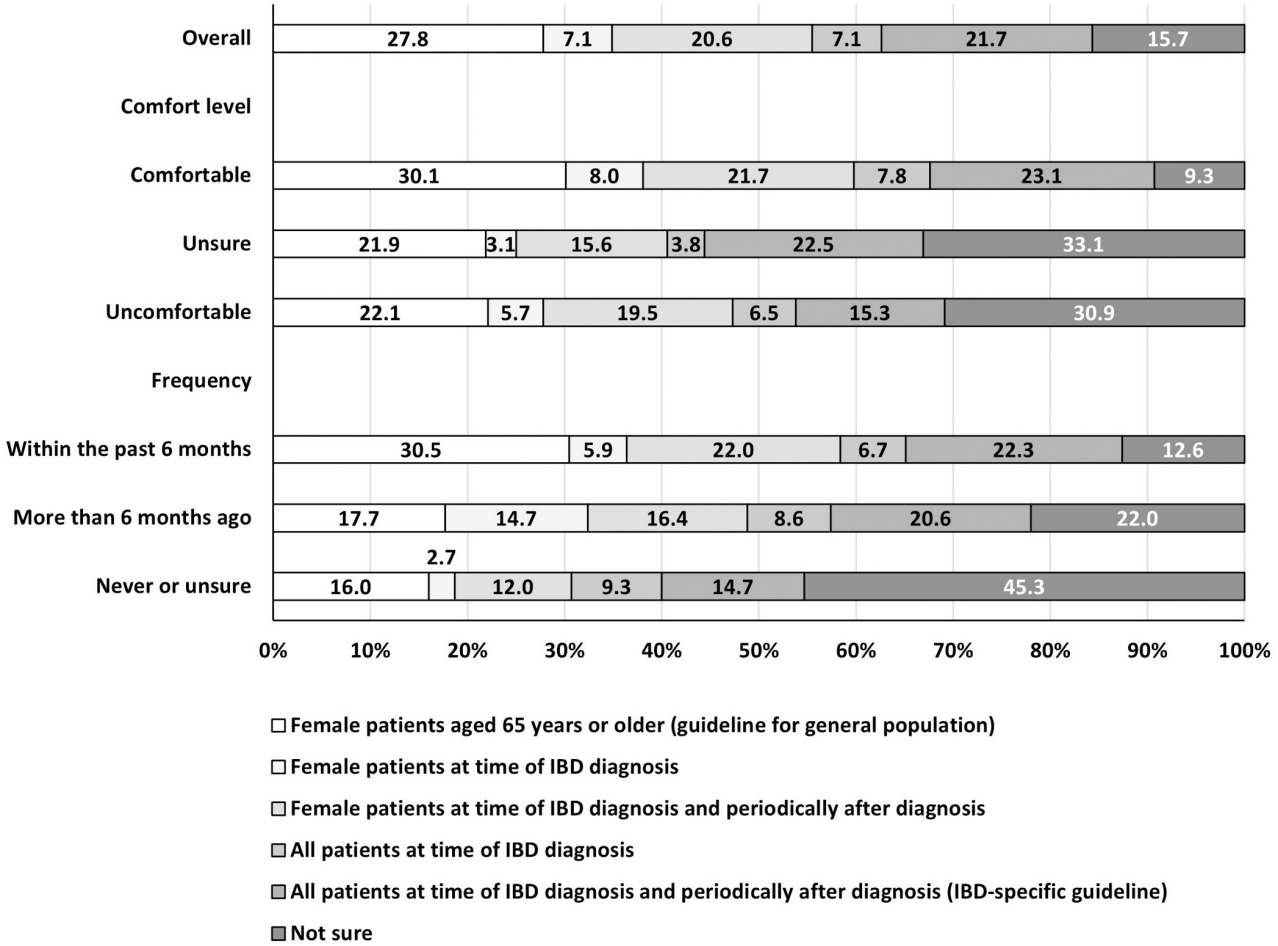
When PCPs would recommend that patients with IBD receive osteoporosis screening with bone mineral density testing,^a^ by comfort level^b^ and frequency^c^ of seeing patients with IBD in practice, DocStyles spring 2022 (*n* = 1,503). Abbreviations: IBD, inflammatory bowel disease; PCPs, primary care providers. ^a^PCPs were asked, “When would you recommend that patients with IBD who have conventional risk factors for abnormal bone mineral density receive an osteoporosis screening with bone mineral density testing?”. ^b^Comfort level is a response to the question, “Are you comfortable or uncomfortable recommending or providing preventive screenings to patients with IBD?”. ^c^Frequency is a response to the question, “In your practice, have you seen patients with inflammatory bowel disease (IBD), a disease which mainly includes Crohn’s disease and ulcerative colitis?”.

**Fig 3:**
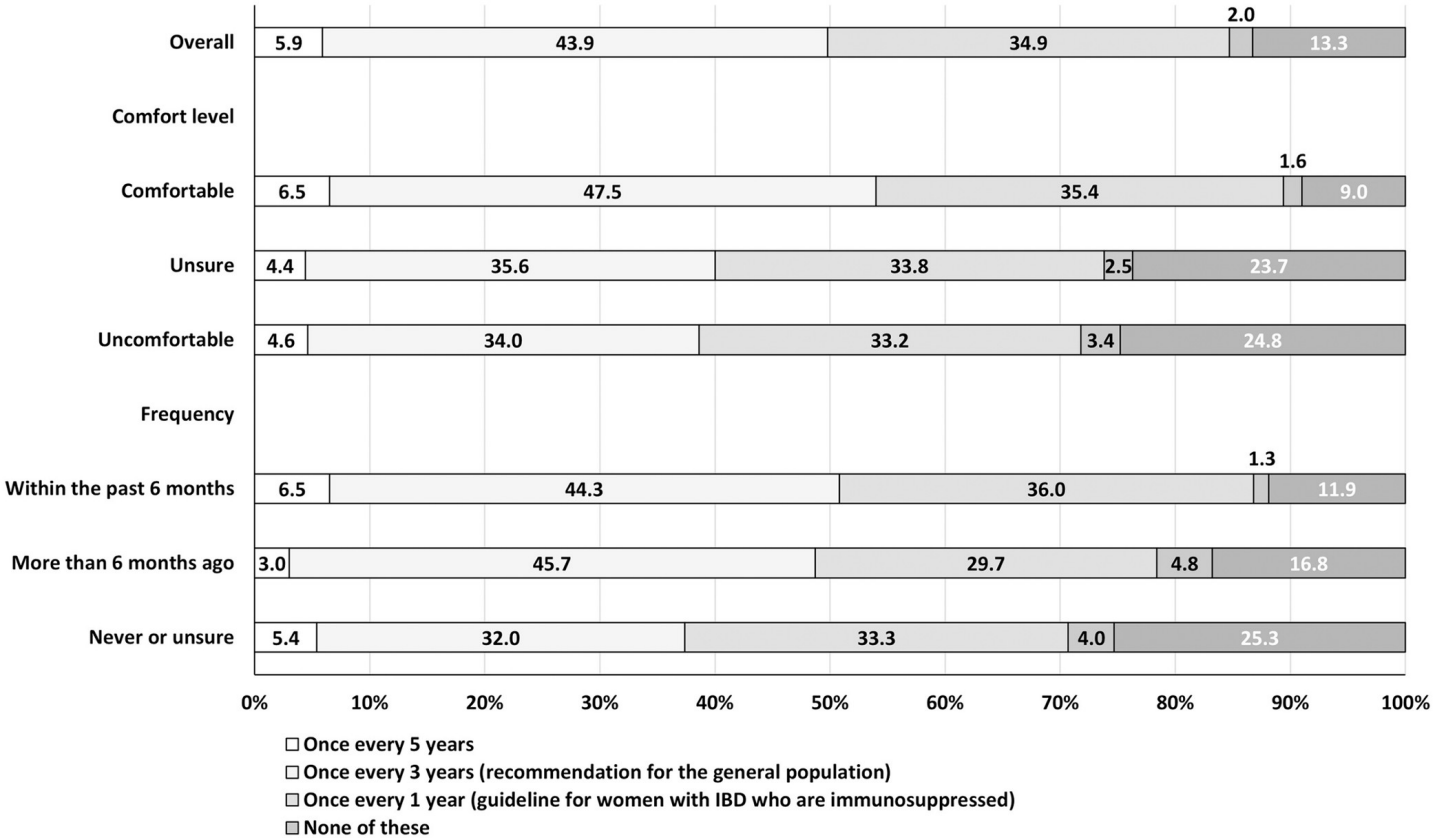
How frequently PCPs would recommend Pap tests for women with IBD on immunosuppressive therapy,^a^ by comfort level^b^ and frequency^c^ of seeing patients with IBD in practice, DocStyles spring 2022 (*n* = 1,503). Abbreviations: IBD, inflammatory bowel disease; PCPs, primary care providers. ^a^PCPs were asked, “How frequently would you recommend Pap tests be initially done (i.e., prior to consecutive normal test results) for women with IBD on immunosuppressive therapy?”. ^b^Comfort level is a response to the question, “Are you comfortable or uncomfortable recommending or providing preventive screenings to patients with IBD?”. ^c^Frequency is a response to the question, “In your practice, have you seen patients with inflammatory bowel disease (IBD), a disease which mainly includes Crohn’s disease and ulcerative colitis?”.

When asked about when they recommend osteoporosis and cervical cancer screenings for patients with IBD, PCPs were more likely to select responses aligned with the USPSTF recommendation for the general population than those aligned to the ACG IBD-specific guidelines (Figs 2 and 3). For osteoporosis screening, 28% of PCPs responded that patients with IBD who have conventional risk factors for abnormal bone mineral density receive such screening when female patients are aged ≥ 65 years (USPSTF recommendation), while 22% responded that all patients should be screened at time of IBD diagnosis and periodically thereafter (ACG IBD-specific guideline). For cervical cancer screening, 44% of PCPs responded that they would recommend Pap tests be initially done for women with IBD on immunosuppressive therapy once every 3 years (USPSTF recommendation), while 35% selected once every year (ACG IBD-specific guideline). The percentage whose responses aligned with IBD-specific osteoporosis and cervical cancer guidelines did not differ by comfort level or frequency of seeing patients with IBD. For both screenings, PCPs who reported being comfortable—vs. those unsure or uncomfortable—were more likely to select responses aligned with the guidelines for the general population. For osteoporosis screening, PCPs who reported seeing patients with IBD frequently—vs. those who infrequently or never saw patients with IBD or were unsure—were more likely to select the response aligned with the USPSTF recommendation for the general population. PCPs who reported being unsure or uncomfortable, or who never saw patients with IBD or were unsure, were more likely to select “unsure” for both questions than their counterparts.

Regarding clinical practice methods used for screening immunosuppressed (e.g., IBD) patients, 57% of PCPs selected timely review of immunosuppressed-specific preventive care guidelines, 40% selected decision support using patients’ electronic health record alerts, and 46% selected clinical risk assessment tools (Table 5). PCPs who used the approach of timely review of immunosuppressed-specific preventive care guidelines—vs. their counterparts—had twice the odds of providing or recommending depression and anxiety screenings and 72% higher odds for skin cancer screening. In addition, the odds of these PCPs selecting responses aligned with IBD-specific ACG screening guideline for osteoporosis and cervical cancer screenings were 59% and 45% higher than their counterparts, respectively. PCPs who used decision support from patients’ electronic health records alerts had 47% higher odds for screening for depression and anxiety than those who did not use this approach. PCPs who used clinical risk assessment tools had 33% higher odds of selecting responses aligned with the ACG guideline for osteoporosis screening than those who did not use this approach.

**Table 5:** Percentages and adjusted odds ratios of PCPs’ likelihood to recommend or provide screenings, or recommend screening in line with ACG screening guidelines, for patients with IBD, by clinical practice methods used for screening immunosuppressed patients.

Clinical practice methods^a^	All (*N* = 1,503) %	Likely to provide or recommend screenings for				Responses align with screening guidelines for IBD			
		Depression and anxiety^b^		Skin cancer^c^		Osteoporosis^d^		Cervical cancer^e^	
		%^f^ (95% CI)	AOR^g^ (95% CI)	%^f^ (95% CI)	AOR^g^ (95% CI)	%^f^ (95% CI)	AOR^g^ (95% CI)	%^f^ (95% CI)	AOR^g^ (95% CI)
**Timely review of preventive care guidelines specific to immunosuppressed patients**									
Yes	57.3	84.8*** (82.4−87.2)	1.97*** (1.49−2.61)	62.8*** (59.6−66.1)	1.72*** (1.37−2.15)	25.0*** (22.1−27.9)	1.59*** (1.21−2.08)	38.0** (34.7−41.2)	1.45** (1.15−1.83)
No (ref)	42.7	71.0 (67.5−74.5)	1	46.1 (42.3−50.0)	1	17.3 (14.4−20.2)	1	30.7 (27.1−34.3)	1
**Decision support using patients’ electronic health records alerts**									
Yes	39.8	83.6*** (80.7−86.6)	1.47* (1.10−1.97)	60.0*** (56.1−64.0)	1.25 (1.00−1.57)	22.2 (18.9−25.6)	1.06 (0.81−1.37)	33.1 (29.3−36.9)	0.96 (0.76−1.21)
No (ref)	60.2	75.8 (73.0−78.6)	1	52.8 (49.6−56.1)	1	21.3 (18.7−24.0)	1	36.0 (32.9−39.2)	1
**Clinical risk assessment tools**									
Yes	45.8	82.3*** (79.4−85.1)	1.30 (0.98−1.73)	58.7** (55.0−62.4)	1.16 (0.93−1.45)	23.8 (20.7−27.0)	1.33* (1.02−1.72)	33.0 (29.5−36.5)	0.86 (0.69−1.08)
No (ref)	54.2	76.1 (73.1−79.0)	1	53.1 (49.7−56.6)	1	19.9 (17.1−22.6)	1	36.4 (33.1−39.8)	1

Abbreviations: PCPs, primary care providers; ACG, American College of Gastroenterology; AOR, adjusted odds ratio; CI, confidence interval; IBD, inflammatory bowel disease; ref, referent group.

* 0.01 < *P* ≤ 0.05

** 0.001 < *P* ≤ 0.01

*** *P* < 0.001.

^a^PCPs can choose one or multiple clinical practice methods.

^b^PCPs’ responses of “very likely” or “likely” vs. “unsure,” “unlikely,” or “very unlikely” to provide or recommend a screening for depression and anxiety to patients with IBD.

^c^PCPs’ responses of “very likely” or “likely” vs. “unsure,” “unlikely,” or “very unlikely” to provide or recommend a screening for skin cancer to patients with IBD.

^d^PCPs’ responses of “All patients at time of IBD diagnosis and periodically after diagnosis” vs. the remaining responses to the question, “When would you recommend that patients with IBD who have conventional risk factors for abnormal bone mineral density receive an osteoporosis screening with bone mineral density testing?”.

^e^PCPs’ responses of “Once every 1 year” vs. the remaining responses to the question, “How frequently would you recommend Pap tests be initially done (i.e., prior to consecutive normal test results) for women with IBD on immunosuppressive therapy?” Results do not change appreciably with or without OB-GYN.

^f^Percentage comparisons are based on χ^2^ test.

^g^The multivariable logistic regressions adjusted for clinical practice methods (other than the variable of interest), being comfortable with recommending or providing preventive screenings to patients with IBD, frequency of seeing IBD patients, medical specialty, years of practice, and average number of patients seen per week. Response to “other methods” is not included in the analysis.

The results based on family practitioners and internists only did not change appreciably from those in the entire study sample (S1–S3 Figs, S1 and S2 Tables).

## Discussion

This large-scale survey assessed PCPs’ current attitudes towards preventive screenings for patients with IBD and highlighted several important findings. First, 7 out of 10 PCPs reported being comfortable recommending or providing preventive screenings to patients with IBD, and PCPs who saw patients with IBD more frequently were more likely to report being comfortable. Second, the major reason for PCPs reporting being unsure or uncomfortable recommending screenings was unfamiliarity with screening guidelines for patients with IBD. Third, although a vast majority of PCPs reported being comfortable or saw patients with IBD frequently in practice, findings suggested that PCPs were more likely to make osteoporosis and cervical cancer screening recommendations that align with preventive screening guidelines for the general population than for patients with IBD. Lastly, findings indicated that timely review of preventive care guidelines specific to immunosuppressed patients was associated with PCPs being more likely to recommend or provide screenings to patients with IBD in line with ACG guidelines—which suggests a role for continuing education on updated screening guidelines for immunosuppressed patients.

One encouraging finding is that most PCPs were comfortable recommending or providing preventive screenings to patients with IBD, and that PCPs seeing more patients, including patients with IBD, were more likely to recommend or provide screenings. However, PCPs were more likely to recommend screenings aligned with general patient guidelines than IBD-specific guidelines. Neither PCPs’ comfort level nor their frequency of seeing patients with IBD was correlated with selecting responses that aligned with IBD-specific guidelines for osteoporosis and cervical cancer screening. This finding was consistent with an Australian study in which general practitioners’ knowledge of and attitudes towards IBD were not related to their comfort level [17]. Our findings suggest there is room for improvement in PCPs’ knowledge and awareness of the ACG guidelines, especially how recommended practices (e.g., frequency of screening) may differ for patients with IBD compared to the general population.

The ACG guidelines underscore the importance of screening all patients with IBD for depression and anxiety, as well as skin cancer. About 4 of 5 PCPs were likely to provide or recommend screenings for depression and anxiety to their patients with IBD, which is encouraging because the prevalence of depression and anxiety among IBD patients is higher than that in the general population [5]. However, only 56% of PCPs were likely to provide or recommend skin cancer screenings. The higher prevalence of PCPs being likely to provide or recommend depression and anxiety screenings for patients with IBD, compared to skin cancer screenings, may partially be explained by the similarity in the depression and anxiety recommendations for IBD patients (per ACG guideline) and for the general population (per USPSTF recommendation) [5–7]. PCPs may not be aware that skin cancer screening is recommended for patients with IBD per ACG guideline, while it is not recommended for the general population. Alternatively, PCPs could be aware of the ACG guideline but feel that the low level of evidence does not justify screening. While the ACG guideline is categorized as a strong recommendation, it is based on a low level of evidence, and a recent meta-analysis did not find a significant association between biologic therapy and melanoma skin cancer [18]. PCPs usually refer patients to dermatologists for further examinations if there is a suspicious lesion [19]. A previous study showed that fewer than 1 of 10 patients with IBD accessed dermatologic care [20], possibly due to PCPs’ low awareness of the high risk of melanoma among patients with IBD and nonmelanoma skin cancer among those 50 years or older taking immunomodulators [5]. It is possible that PCPs were less familiar with the screening guidelines specific to patients with IBD. These findings emphasize the importance of continued education on IBD-specific ACG guideline and raising PCPs’ awareness of skin cancer screenings and increasing referral to dermatologists.

Notably, only 1 of 5 PCPs recommended osteoporosis screening for patients with IBD per the ACG guideline. A 2014 Swiss study that used chart reviews to examine osteoporosis diagnostics and treatment in an IBD cohort indicated that the compliance rate of receiving bone mineral density testing was low and identified inconsistent usage of osteoporosis screening practice and underuse of osteoporosis treatment in IBD patients [21]. Although not a direct comparison, the inconsistencies in osteoporosis screening and treatment practices found in this previous study [21] and our study highlight a lack of consistency among practitioners related to osteoporosis screening for adults with IBD. Our findings may also imply that PCPs may not have been familiar with the updated IBD-specific ACG guideline [5] for osteoporosis screening, which is also in alignment with the USPSTF recommendation for women with high-risk factors [10].

Only 1 of 3 PCPs recommended screening women with IBD on immunosuppressive therapy according to the ACG guideline for cervical cancer screening. The ACG guideline for cervical cancer screening aligns with other guidelines for adults and adolescents with HIV infection [22] and immunosuppressed women without HIV infection [23]. Despite these guidelines, women with IBD do not receive adequate screenings, possibly due to lack of physician and patient awareness [24,25]. Although our study showed that OB-GYNs—compared to family practitioners and internists—were less likely to report being comfortable recommending or providing preventive screenings to patients with IBD, they were significantly more likely to select the response to the Pap test question that aligned with the ACG guideline (results not shown). This may be due to OB-GYNs focus on gynecological care. In a survey that assessed who physicians felt was responsible for providing cervical cancer screenings for women with IBD, PCPs were least familiar with the guideline recommending annual screening for immunocompromised women, compared with gastroenterologists and OB-GYNs [26]. Our findings suggest that continuing education to PCPs—especially internists and family practitioners—regarding cervical cancer screening for women with IBD, may help increase the screening or referral rate among this group.

Finally, our study demonstrated that timely review of preventive care guidelines specific to immunosuppressed patients may increase PCPs’ likelihood of providing or recommending screenings to patients with IBD. This finding underscores the importance of continuing education for PCPs to stay updated with preventive screening guidelines, such as the ACG guideline, to improve screening rates. Physicians may have different views related to who should be responsible for preventive care for patients with IBD. For example, in one survey study, OB-GYNs thought that they should be responsible for performing Pap tests for patients with IBD [26]. Another study surveying PCPs and gastroenterologists found that gastroenterologists were more likely than PCPs to perform health maintenance practice for patients with IBD, such as screenings for melanoma and nonmelanoma skin cancers and osteoporosis, whereas PCPs were more likely to assess depression and anxiety than gastroenterologists [27]. The same study also showed that both gastroenterologists and PCPs felt IBD-related health maintenance issues were their own responsibilities [27]. Although there is no consensus as to who should be responsible for preventing screenings, multidisciplinary efforts from medical specialties have shown to deliver the best management outcomes to patients with IBD [28].

The study has at least 4 limitations. First, we were not able to determine other clinical methods or other possible reasons for PCPs’ not feeling comfortable, because the DocStyles survey does not allow for open-ended responses. Second, we were not able to assess gastroenterologists’ comfort level and responses about preventive screenings because DocStyles does not include this medical specialty. Third, for assessing cervical cancer screening, PCPs were asked about Pap tests only; questions about HPV testing were not included. Fourth, the results were based on active members of Sermo’s Global Medical Panel, therefore the findings cannot generalize to all US PCPs. However, the distribution of PCPs’ regions of practice in the 2022 DocStyles survey are comparable to those in the American Medical Association Master file [29]. Fifth, other than the IBD-related questions, PCPs’ clinical characteristics were generic information collected in the overall DocStyles survey which may not be specific to the current study objectives. Sixth, we assessed frequency of seeing patients with IBD in practice which was significantly associated with PCPs’ comfort level. However, the survey did not collect information regarding number of patients with IBD seen in practice which may also be associated with PCPs’ comfort level. Despite the limitations, the survey included a large sample and reflected current PCPs’ attitudes towards recommending and providing preventive screenings to patients with IBD and the findings will inform primary care practice.

## Conclusions

A vast majority of PCPs in this survey study reported being comfortable recommending or providing preventive screenings to patients with IBD, although the reported practices for osteoporosis and cervical cancer did not align well with the IBD-specific ACG guideline. Continued efforts and strategies are needed to improve PCPs’ awareness to deliver timely and appropriate preventive care to patients with IBD.

## Supporting information

S1 Checklist (DOCX)

S1 Fig Reasons identified by family practitioners and internists who are uncomfortable or unsure of recommending or providing preventive screenings to patients with IBD, DocStyles spring 2022 (*n* = 207)^a^. Abbreviations: IBD, inflammatory bowel disease. ^a^The multiple-choice question, “What are the reasons that you are unsure or uncomfortable?” applied only to respondents who answered “uncomfortable” or “unsure” to the question, “Are you comfortable or uncomfortable recommending or providing preventive screenings to patients with IBD?”.
(TIF)

S2 Fig When family practitioners and internists would recommend that patients with IBD receive osteoporosis screening with bone mineral density testing,^a^ by comfort level^b^ and frequency^c^ of seeing patients with IBD in practice, DocStyles spring 2022 (*n* = 1,000). Abbreviations: IBD, inflammatory bowel disease. ^a^Family practitioners and internists were asked, “When would you recommend that patients with IBD who have conventional risk factors for abnormal bone mineral density receive an osteoporosis screening with bone mineral density testing?”. ^b^Comfort level is a response to the question, “Are you comfortable or uncomfortable recommending or providing preventive screenings to patients with IBD?”. ^c^Frequency is a response to the question, “In your practice, have you seen patients with inflammatory bowel disease (IBD), a disease which mainly includes Crohn’s disease and ulcerative colitis?”.
(TIF)

S3 Fig How frequently family practitioners and internists would recommend Pap tests for women with IBD on immunosuppressive therapy,^a^ by comfort level^b^ and frequency^c^ of seeing patients with IBD in practice, DocStyles spring 2022 (*n* = 1,000). Abbreviations: IBD, inflammatory bowel disease. ^a^Family practitioners and internists were asked, “How frequently would you recommend Pap tests be initially done (i.e., prior to consecutive normal test results) for women with IBD on immunosuppressive therapy?”. ^b^Comfort level is a response to the question, “Are you comfortable or uncomfortable recommending or providing preventive screenings to patients with IBD?”. ^c^Frequency is a response to the question, “In your practice, have you seen patients with inflammatory bowel disease (IBD), a disease which mainly includes Crohn’s disease and ulcerative colitis?”.
(TIF)

S1 Table Percentages and adjusted odds ratios of family practitioners and internists’ likelihood^a^ to provide or recommend screenings for depression, anxiety and skin cancer by comfort level, and frequency of seeing patients with IBD in practice/seeing patients with IBD in practice. (DOCX)

S2 Table Percentages and adjusted odds ratios of family practitioners and internists’ likelihood to recommend or provide screenings, or recommend screening in line with ACG screening guidelines, for patients with IBD, by clinical practice methods used for screening immunosuppressed patients. (DOCX)
