# Supplementary figures and images for: Understanding primary care providers’ attitudes towards preventive screenings to patients with inflammatory bowel disease
Source: PLoS One. 2024 Apr 25;19(4):e0299890. doi: 10.1371/journal.pone.0299890 (PMC11045111; doi:10.1371/journal.pone.0299890)

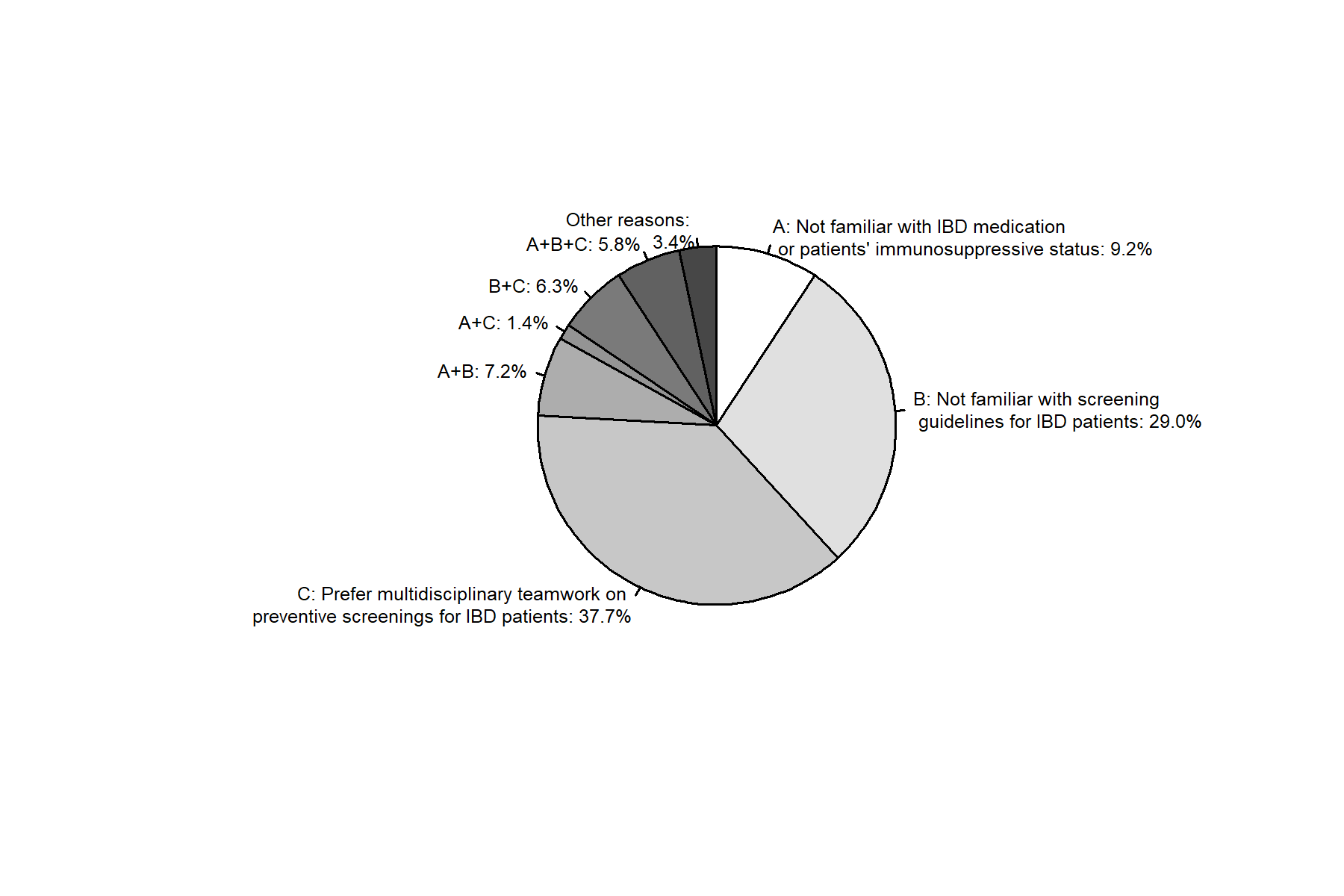

Supplement: S1 Fig — Abbreviations: IBD, inflammatory bowel disease. aThe multiple-choice question, “What are the reasons that you are unsure or uncomfortable?” applied only to respondents who answered “uncomfortable” or “unsure” to the question, “Are you comfortable or uncomfortable recommending or providing preventive screenings to patients with IBD?”. (TIF) [file pone.0299890.s002.tif]

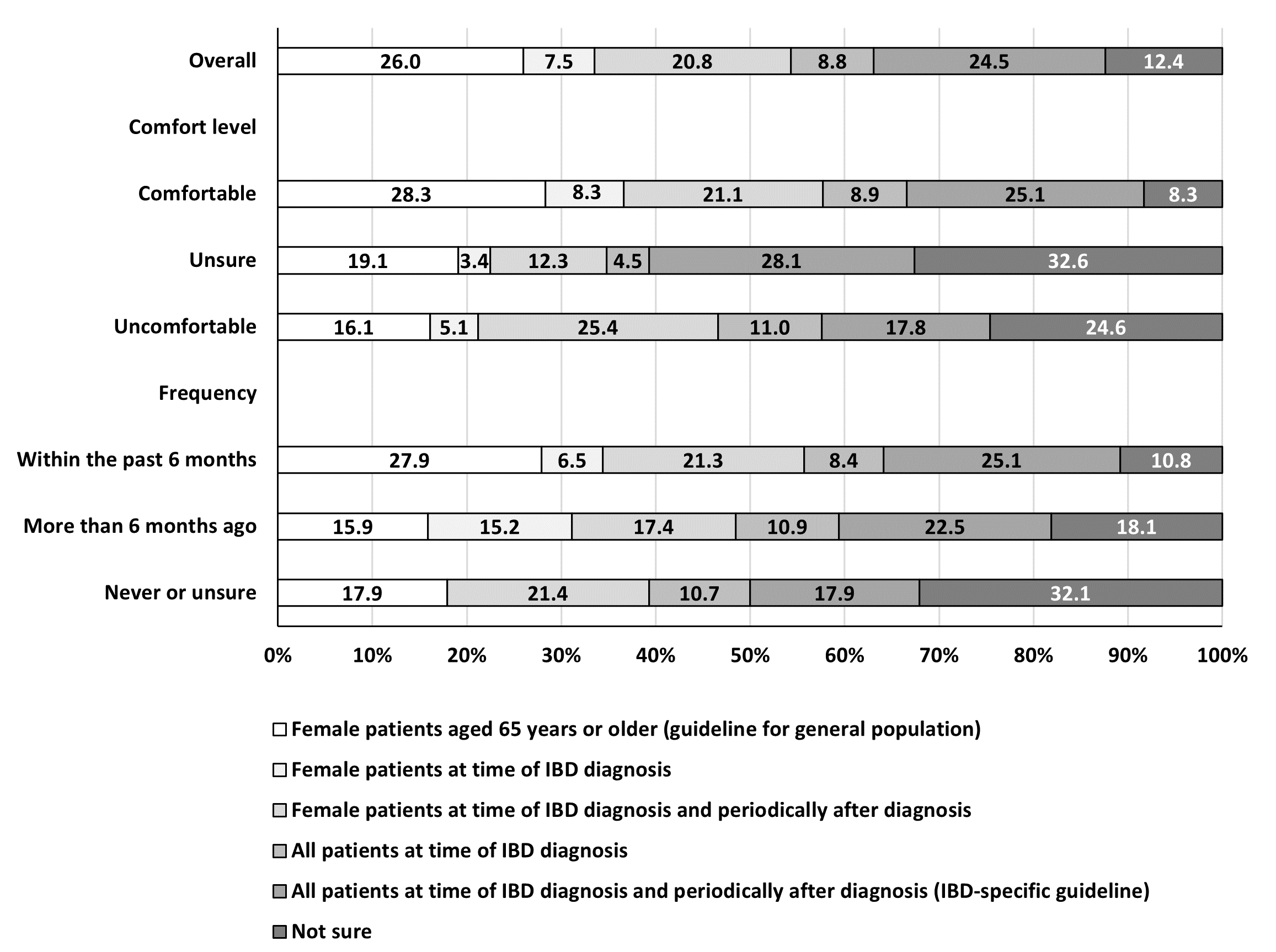

Supplement: S2 Fig — Abbreviations: IBD, inflammatory bowel disease. aFamily practitioners and internists were asked, “When would you recommend that patients with IBD who have conventional risk factors for abnormal bone mineral density receive an osteoporosis screening with bone mineral density testing?”. bComfort level is a response to the question, “Are you comfortable or uncomfortable recommending or providing preventive screenings to patients with IBD?”. cFrequency is a response to the question, “In your practice, have you seen patients with inflammatory bowel disease (IBD), a disease which mainly includes Crohn’s disease and ulcerative colitis?”. (TIF) [file pone.0299890.s003.tif]

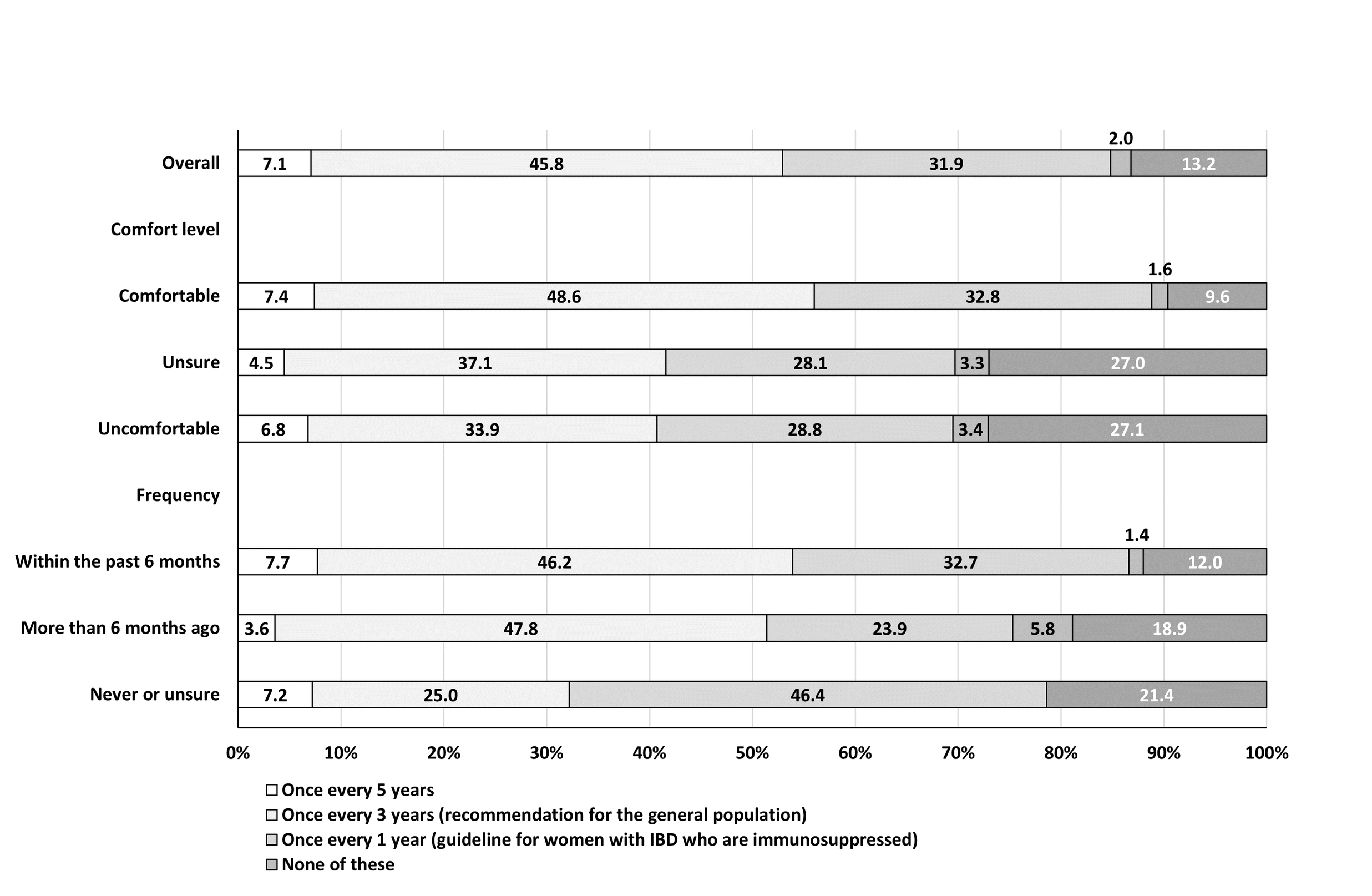

Supplement: S3 Fig — Abbreviations: IBD, inflammatory bowel disease. aFamily practitioners and internists were asked, “How frequently would you recommend Pap tests be initially done (i.e., prior to consecutive normal test results) for women with IBD on immunosuppressive therapy?”. bComfort level is a response to the question, “Are you comfortable or uncomfortable recommending or providing preventive screenings to patients with IBD?”. cFrequency is a response to the question, “In your practice, have you seen patients with inflammatory bowel disease (IBD), a disease which mainly includes Crohn’s disease and ulcerative colitis?”. (TIF) [file pone.0299890.s004.tif]
