# Supplementary material for: Understanding primary care providers’ attitudes towards preventive screenings to patients with inflammatory bowel disease
Source: PLoS One. 2024 Apr 25;19(4):e0299890. doi: 10.1371/journal.pone.0299890 (PMC11045111; doi:10.1371/journal.pone.0299890)
Supplement: S1 Table — (DOCX) [file pone.0299890.s005.docx]

**S1 Table. Percentages and adjusted odds ratios of family practitioners and internists’ likelihood^a^ to provide or recommend screenings for depression, anxiety and skin cancer by comfort level, and frequency of seeing patients with IBD in practice/seeing patients with IBD in practice**

| **Select characteristics** | **All**  **(*N* = 1,000)**  **%** | **Likely to provide or recommend screenings for** | | | |
| --- | --- | --- | --- | --- | --- |
|  |  | **Depression and anxiety** | | **Skin cancer** | |
|  |  | **%^b^**  **(95% CI)** | **AOR**  **(95% CI)** | **%^b^**  **(95% CI)** | **AOR**  **(95% CI)** |
| **All** | 100 | 81.9 (79.5−84.3) | -- | 61.7  (58.7−64.7) | -- |
| **Reported level of comfort recommending or providing preventive screenings^c^** | | | | | |
| Comfortable | 79.3 | 86.9*** (84.5−89.2) | 3.63*** (2.33−5.66) | 67.1*** (63.8−70.4) | 3.43***  (2.25−5.21) |
| Unsure | 8.9 | 66.3 (56.5−76.1) | 1.31 (0.72−2.38) | 48.3  (37.9−58.7) | 1.74 (0.98−3.07) |
| Uncomfortable (ref) | 11.8 | 60.2  (51.3−69.0) | 1 | 35.6 (27.0−44.2) | 1 |
| **Reported frequency of seeing IBD patients in practice^d^** | | | | | |
| Within the past 6 months | 83.4 | 85.0*** (82.6−87.4) | 2.01*** (1.34−3.01) | 63.8**  (60.5−67.1) | 1.28 (0.89−1.83) |
| More than 6 months ago or never or not sure (ref)^e^ | 16.6 | 66.3  (59.1−73.5) | 1 | 51.2  (43.6−58.8) | 1 |

* 0.01 < *P* ≤ 0.05 ** 0.001 < *P* ≤ 0.01 *** *P* < 0.001.

Abbreviations: AOR, adjusted odds ratio; CI, confidence interval; IBD, inflammatory bowel disease; ref, referent group.

^a^ Family practitioners and internists were asked how likely they were to provide or recommend a screening for “depression and anxiety” or “skin cancer” to patients with IBD. Responses were “very likely” or “likely” vs. “neither likely nor unlikely,” “unlikely,” or “very unlikely.”

^b^ Percentage comparisons are based on χ^2^ test.

^c^The multivariable logistic regressions adjusted for frequency of seeing IBD patients, medical specialty, years of practice, and average number of patients seen per week.

^d^The multivariable logistic regressions adjusted for being comfortable recommending or providing preventive screenings to IBD patients, medical specialty, years of practice, and average number of patients seen per week.

^e^Due to the small sample size of “not sure” and “never”, “more than 6 months ago”, these three categories were combined.
