# Supplementary material for: Understanding primary care providers’ attitudes towards preventive screenings to patients with inflammatory bowel disease
Source: PLoS One. 2024 Apr 25;19(4):e0299890. doi: 10.1371/journal.pone.0299890 (PMC11045111; doi:10.1371/journal.pone.0299890)
Supplement: S2 Table — (DOCX) [file pone.0299890.s006.docx]

**S2 Table. Percentages and adjusted odds ratios of family practitioners and internists’ likelihood to recommend or provide screenings, or recommend screening in line with ACG screening guidelines, for patients with IBD, by clinical practice methods used for screening immunosuppressed patients**

| **Clinical practice methods^a^** | **All**  **(*N* = 1,000)**  **%** | **Likely to provide or recommend screenings for** | | | | **Responses align with screening guidelines for IBD** | | | |
| --- | --- | --- | --- | --- | --- | --- | --- | --- | --- |
|  |  | **Depression and anxiety^b^** | | **Skin cancer^c^** | | **Osteoporosis^d^** | | **Cervical cancer^e^** | |
|  |  | **%^f^**  **(95% CI)** | **AOR^g^**  **(95% CI)** | **%^f^**  **(95% CI)** | **AOR^g^**  **(95% CI)** | **%^f^**  **(95% CI)** | **AOR^g^**  **(95% CI)** | **%^f^**  **(95% CI)** | **AOR^g^**  **(95% CI)** |
| **Timely review of preventive care guidelines specific to immunosuppressed patients** | | | | | | | | |  |
| Yes | 62.6 | 86.7***  (84.1−89.4) | 2.05*** (1.43−2.94) | 67.4*** (63.7−71.1) | 1.75***  (1.32−2.32) | 27.3**  (23.8−30.8) | 1.58**  (1.14−2.19) | 35.5** (31.7−39.2) | 1.56** (1.16−2.11) |
| No (ref) | 37.4 | 73.8  (69.3−78.3) | 1 | 52.1 (47.1−57.2) | 1 | 19.8 (15.8−23.8) | 1 | 25.9 (21.5−30.4) | 1 |
| **Decision support using patients’ electronic health records alerts** | | | | | | | | | |
| Yes | 41.1 | 85.9** (82.5−89.3) | 1.53* (1.06−2.22) | 66.2*  (61.6−70.8) | 1.35*  (1.02−1.78) | 25.6 (21.3−29.8) | 1.16 (0.85−1.58) | 29.7 (25.3−34.1) | 0.97 (0.73−1.30) |
| No (ref) | 58.9 | 79.1 (75.8−82.4) | 1 | 58.6 (54.6−62.6) | 1 | 23.8 (20.3−27.2) | 1 | 33.5 (29.6−37.3) | 1 |
| **Clinical risk assessment tools** | | | | | | | | | |
| Yes | 44.9 | 84.0 (80.6−89.3) | 1.16 (0.81−1.66) | 63.7 (59.3−68.2) | 1.04  (0.79−1.36) | 26.3  (22.2−30.4) | 1.22 (0.90−1.65) | 29.0 (24.8−33.2) | 0.84 (0.64−1.12) |
| No (ref) | 55.1 | 80.2  (76.9−83.5) | 1 | 60.1 (56.0−64.2) | 1 | 23.1 (19.5−26.6) | 1 | 34.3 (30.3−38.3) | 1 |

Abbreviations: ACG, American College of Gastroenterology; AOR, adjusted odds ratio; CI, confidence interval; IBD, inflammatory bowel disease; ref, referent group.

* 0.01 < *P* ≤ 0.05 ** 0.001 < *P* ≤ 0.01 *** *P* < 0.001.

^a^Family practitioners and internists can choose one or multiple clinical practice methods.

^b^Responses of “very likely” or “likely” vs. “unsure,” “unlikely,” or “very unlikely” to provide or recommend a screening for depression and anxiety to patients with IBD from family practitioners and internists.

^c^Responses of “very likely” or “likely” vs. “unsure,” “unlikely,” or “very unlikely” to provide or recommend a screening for skin cancer to patients with IBD from family practitioners and internists.

^d^Responses of “All patients at time of IBD diagnosis and periodically after diagnosis” vs. the remaining responses to the question, “When would you recommend that patients with IBD who have conventional risk factors for abnormal bone mineral density receive an osteoporosis screening with bone mineral density testing?” from family practitioners and internists.

^e^Responses of “Once every 1 year” vs. the remaining responses to the question, “How frequently would you recommend Pap tests be initially done (i.e., prior to consecutive normal test results) for women with IBD on immunosuppressive therapy?” from family practitioners and internists. Results do not change appreciably with or without OB-GYN.

^f^Percentage comparisons are based on χ^2^ test.

^g^The multivariable logistic regressions adjusted for clinical practice methods (other than the variable of interest), being comfortable with recommending or providing preventive screenings to patients with IBD, frequency of seeing IBD patients, medical specialty, years of practice, and average number of patients seen per week. Response to “other methods” is not included in the analysis.
